# PhageTerm: a tool for fast and accurate determination of phage termini and packaging mechanism using next-generation sequencing data

**DOI:** 10.1038/s41598-017-07910-5

**Published:** 2017-08-15

**Authors:** Julian R. Garneau, Florence Depardieu, Louis-Charles Fortier, David Bikard, Marc Monot

**Affiliations:** 1 Département de Microbiologie, Institut Pasteur, Laboratoire Pathogenèse des bactéries anaérobies, Paris, France; 2 Département de Microbiologie et d’infectiologie, Faculté de Médecine et des Sciences de la Santé, Université de Sherbrooke, Sherbrooke, QC Canada; 3 Département de Microbiologie, Institut Pasteur, Groupe Biologie de Synthèse, Paris, France; 4 Université Paris Diderot, Sorbonne Paris Cité, Paris, France

## Abstract

The worrying rise of antibiotic resistance in pathogenic bacteria is leading to a renewed interest in bacteriophages as a treatment option. Novel sequencing technologies enable description of an increasing number of phage genomes, a critical piece of information to understand their life cycle, phage-host interactions, and evolution. In this work, we demonstrate how it is possible to recover more information from sequencing data than just the phage genome. We developed a theoretical and statistical framework to determine DNA termini and phage packaging mechanisms using NGS data. Our method relies on the detection of biases in the number of reads, which are observable at natural DNA termini compared with the rest of the phage genome. We implemented our method with the creation of the software PhageTerm and validated it using a set of phages with well-established packaging mechanisms representative of the termini diversity, i.e. 5′*cos* (Lambda), 3′*cos* (HK97), *pac* (P1), headful without a *pac* site (T4), DTR (T7) and host fragment (Mu). In addition, we determined the termini of nine *Clostridium difficile* phages and six phages whose sequences were retrieved from the Sequence Read Archive. PhageTerm is freely available (https://sourceforge.net/projects/phageterm), as a Galaxy ToolShed and on a Galaxy-based server (https://galaxy.pasteur.fr).

## Introduction

Bacteriophages (phages), the viruses of Bacteria, come in a diversity of shapes and sizes^1^. They all produce virion particles consisting of the genetic material surrounded by a protein shell (capsid), sometimes with the presence of an intervening lipid membrane. Their nucleic acid content can be double stranded DNA (dsDNA), single-stranded DNA, double-stranded RNA or single-stranded RNA. Different conformations of the nucleic acid also exist with some phage genomes being encapsidated as single-stranded circular molecules and others as double-stranded linear molecules. The vast majority of phages described to date produce capsids containing linear dsDNA, but even when considering these phages only, an impressive diversity of packaging mechanisms has been described, leading to various type of DNA termini^2^.

Towards the end of their infection cycle, dsDNA phages generally form concatemers that are cut by the terminase during packaging to form the mature chromosome^3^. There are four main mechanisms used by phages to recognize their own DNA (rather than their host’s DNA) and initiate and then terminate its packaging: (i) The terminase can recognize a specific site where it introduces a staggered cut (*cos* site), thereby generating fixed DNA termini with cohesive ends that can either have 5′ or 3′ overhangs (e.g. Lambda^4^, HK97^5^). (ii) A fixed position can be recognized on the phage DNA where direct terminal repeats (DTR) will be generated by extension synthesis at the 3′ ends of staggered nicks. The size of these DTRs can range from just over a hundred bases (e.g. T3^6^, T7^7^) to more than ten thousand bases (e.g. T5^8^, Spo1^9^). Phage N4 carries terminal repeats with an accurate terminus on the left end but several possible termini on the right end^10^. (iii) The terminase can initiate packaging on the phage concatemer at a specific packaging site (*pac* site), and the subsequent cuts are made at variable positions, when the phage head becomes full (e.g. P1^11^, P22^12^). This leads to capsids containing circularly permutated genomes with redundant ends used to circularize the phage genome through recombination after injection in the host cell. (iv) T4-like phages use a variant of this headful packaging mechanism in which no *pac* site is recognized and packaging is rather initiated randomly^13^. These phages usually degrade the host DNA, ensuring that only viral DNA is packaged.

Three less-frequent packaging strategies that do not involve the formation of concatemers must also be considered: (i) Phage P2 carries a *cos* site, but the packaging substrate is circular dsDNA^14^. (ii) Phage Mu replicates through transposition in the host genome and carries pieces of the host DNA as its termini^15^. (iii) The Bacillus phage phi29 carries covalently-bound proteins at its DNA termini^16^. When considering the small number of phages for which the termini have been precisely studied, it is likely that yet other packaging mechanisms exist in nature.

In this study, we investigated how the information gathered by high-throughput sequencing approaches, in particular Illumina technologies, can be used to predict the DNA termini and packaging mechanisms of dsDNA phages. The experimental procedures traditionally used for this purpose rely on the identification and cloning of restriction fragments containing the termini. This can be delicate and cumbersome, especially in the case of circularly-permutated phages, for which the packaging sites are located within sub-molar fragments after digestion^17^. For certain types of termini, traditional procedures of identification require additional work to retrieve the packaging orientation, the exact position of the termini, and the cohesive sequence.

**Figure 1:**
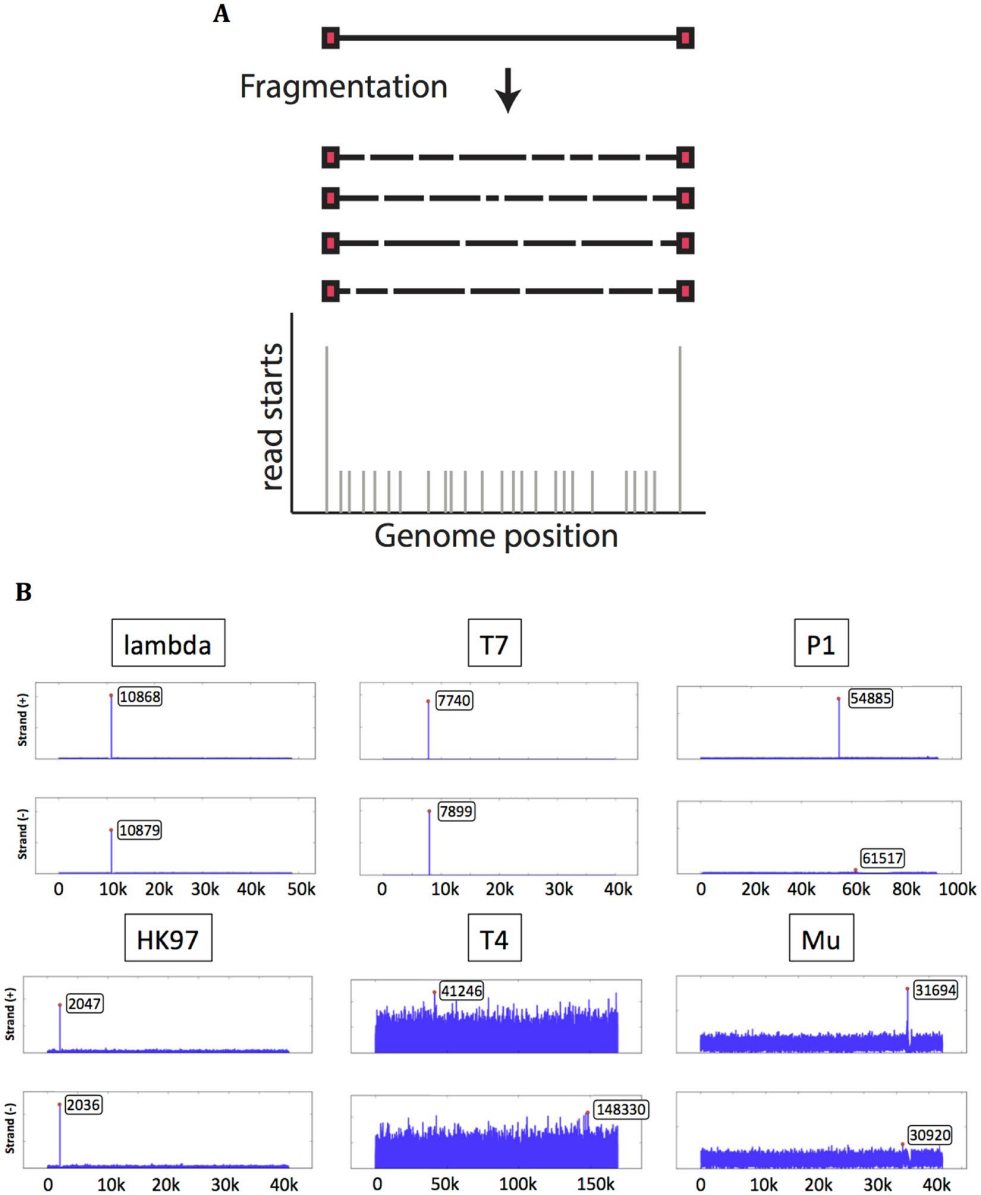
Biases in the number of reads starting at DNA termini. (**A**) After fragmentation, natural termini are present once per phage genome, while DNA ends produced by fragmentation will fall at random positions along the genome. The black line represents the phage genome, the red squares are the phage termini. (**B**) Starting position coverage for each strand of six reference phages (Lambda, HK97, T7, P1, T4, Mu).

Many high-throughput sequencing methods rely on the random fragmentation of DNA, followed by repair of DNA ends and adapter ligation. After the fragmentation process, natural DNA termini are normally present once per phage genome, while DNA ends produced by fragmentation are scattered randomly along the genome (Fig. 1A). This means that significantly more reads start at the phage termini position than anywhere else in the genome. This observation was made by several groups, and was recently used by Li *et al*.^18^ to characterize the DNA termini of several phages. The method used in this study, however, did not consider several common packaging modes, relied on arbitrary thresholds to classify the phage packaging mechanisms, and was not robust enough to handle poor data quality or uneven coverage.

We propose here a theoretical and statistical framework to robustly analyze DNA termini and phage packaging mechanisms using randomly-fragmented next-generation sequencing data. Our methods are implemented in the PhageTerm software which we have made freely available. A graphical user interface is also available as a Galaxy wrapper at https://galaxy.pasteur.fr and can be installed through the Galaxy public ToolShed repository.

## Methods

### PhageTerm Analysis

Sequencing reads were mapped on the reference to determine the starting position coverage (SPC) as well as the coverage (COV) in each orientation. The fragment coverage was computed by considering that all the bases between a pair of reads were covered. These values were then used to compute the variable *tau*: *τ* = *SPC*/*COV*. This variable has several interesting properties that were useful to determine DNA termini. We recommend inputting paired-end sequencing information when using PhageTerm to enable the computation of fragment coverage, but most analysis can still be performed using single reads. In that case, sequence coverage is computed as an approximation of fragment coverage but with the caveat that the value of *τ* becomes more complex to interpret (see below).

#### *Cos* phages

The coverage at position *i* (COVi) is determined by the number of fragments that start at position *i* (SPCi) plus the number of fragments that start before *i* and cover *i*. In the case of a fixed DNA terminus no reads should start before the terminus. Therefore $COVi=SPCi$ and *τ* = 1. Note that different outcomes are expected for *cos* phages, depending on the type of termini, i.e. 5′ or 3′ cohesive ends. The end-repair enzymes typically used during sequencing library preparation fill 5′ overhangs and cut 3′ overhangs. A value of *τ* = 1 is thus expected for 3′*cos* phages. However for 5′*cos* phages, end-repair produced overlapping ends: fragments that ended at the right terminus are seen as covering the left terminus and conversely. The expected value of *τ* was thus 0.5.

#### DTR phages

In the case of DTR phages, for N phage particles in a sample that undergo fragmentation, there should be N fragments that start at the terminus, and N fragments that cover the edge of the repeat on the other side of the genome. As a result, *τ* is expected to be 0.5.

#### *Pac* phages

In the case of *pac* phages, for N phages present in the sample, there should be *N*/*C* fragments starting at the *pac* site, where C is the number of phage genome copies per concatemer. In the same sample, N fragments should cover the *pac* site position. Therefore, at the *pac* site position, *τ* is expected to be:

1 $$\,\tau =(\frac{{\boldsymbol{N}}}{{\boldsymbol{C}}})/({\boldsymbol{N}}+\frac{{\boldsymbol{N}}}{{\boldsymbol{C}}})=1/(1+{\boldsymbol{C}})$$

The number of phage genome copies per concatemer (C) reported in the literature is typically smaller than 10^19^ and therefore 0.1 < *τ* < 0.5.

### Single-read versus paired-end sequencing

Note that when single reads are used, sequence coverage is computed instead of fragment coverage, which can affect the expected value of *τ*. In the case of *cos* phages, if the average fragment length is smaller than the read length, then few to no forward reads are obtained that start before and cover the left terminus. As a consequence, the expected value of *τ* at the left terminus is 1. The same argument holds in the other orientation. In the case of DTR phages, when the read length is smaller than the average fragment length minus the DTR size, then few to no forward reads are obtained that start before and cover the edge of right repeat. This will frequently be the case when sequencing phages with short DTR. As a result, a value of *τ* as high as 1 can be obtained. The same argument holds in the other orientation.

### Calling significant termini

Another interesting property of *τ* is that its average value at positions along the genome that are not termini is expected to be 1/*F*, where F is the average fragment size, which can be easily computed from paired-end sequencing data. Indeed, if the average number of reads that start at a random position was $\overline{SPC}=R$, then the average number of fragments that cover random positions is $\overline{COV}$ = *R* * *F* and $\bar{\tau }=1/F$. The average fragment size in Illumina sequencing libraries usually ranges from $300 < F < 800bp$, so $\bar{\tau }$ < 3 * 10^−3^. Note that $\bar{\tau }$ should always be much smaller than the expected value of *τ* at termini position in any of the situations described where $\,0.1\le \tau \le 1$. Therefore, peaks in the value $\tau$ at termini position can be detected easily in most cases. Nonetheless, calling the position of *pac* sites where *τ* < 0.5 can be made more difficult because of the experimental noise introduced in the many steps of library preparation.

To assess whether the number of reads starting at a given position along the genome can be considered a significant outlier, PhageTerm first segments the genome according to coverage using a regression tree. This allows a robust analysis to be performed even in the case of incorrect assemblies where the coverage along the reference sequence might be variable. A gamma distribution is then fitted to SPC values for each segment and an adjusted p-value is computed for each position. A significance threshold of 0.01 was chosen to consider that a peak in *τ* represents a valid terminus. Finally, if several peaks with an adjusted p-value lower than 1/*G* (phage genome length) were detected within a small sequence window (default: 20 bp), the position was deemed significant. This allows detecting packaging sites for which the terminase can cleave at several nearby positions. In such cases, the number of reads starting at nearby peaks was added for subsequent analysis, and the position of the highest peak was reported.

### Phage classification

The following rules were then applied to classify the phages: when significant peaks with *τ* > 0.1 were found on both strands, the distance between the peaks was used to differentiate *cos* phages and DTR phages. To our knowledge, the largest cohesive ends described are 19 bases long, reported for phage P2^17, 20^, and the smallest DTR, 131 bases from phage T3. We therefore placed a threshold at 20 bp. An additional criterion to classify a phage as DTR was that the coverage between the peaks should be at least 10% higher than the average coverage. Phages classified as *cos* were predicted to have 5′ overhangs if the forward terminus was to the left of the reverse terminus, and 3′ overhangs otherwise. When a significant peak with *τ* > 0.1 was found only on one strand, the phage was considered to be a *pac* phage. PhageTerm also provides the orientation of packaging for *pac* phages, as determined by the strand carrying the significant peak. PhageTerm returns “Multiple” for phages that contain more than one significant peak on the same strand without *τ* > 0.35.

Phages for which no significant peaks were found might either use a headful packaging mechanism without a preferred packaging site (T4-like), or be Mu-like phages. To differentiate both possibilities, the software computes the number of fragments for which one read matches the host and the other read matches the phage genome for paired-ends, or each side of the same read for single-reads sequencing. Phages in the sample were, on average, fragmented into *G*/*F* pieces where F is the average fragment size and G the size of the phage genome. In the case of Mu-like phages, out of *G*/*F* phage fragments, two should be hybrid fragments. The proportion of hybrid fragments is thus expected to be 2 * (*F*/*G*). For single-ends sequencing, hybrid fragment size is replaced by the read length. Note that these fragments or reads were only detected if the region spanning the host genome and the phage genome were both longer than the seed sequence (S) used to align the reads. The theoretical expectation of the proportion of hybrid fragments can thus be corrected as 2 * (*F* − 2 * *S*)/*G*. PhageTerm assigned the Mu-like class if the proportion of hybrid fragments or reads was at least half the expected value. The position of hybrid reads on the phage genome was then used to estimate the termini positions. Finally, phages for which no hybrid fragments or reads were found were classified as unknown.

Lower than expected *τ* values could indicate that the phage DNA was contaminated with unpackaged DNA. In the case where this can be excluded, *τ* values that deviate from the expectations might indicate novel packaging mechanisms.

Some additional information can also be extracted depending on phage characteristics: (i) an estimation of concatemer size for *pac* phages; and (ii) the *cos* or DTR sequence located between the two termini. The output of the two methods are consolidated in a detailed PDF report produced at the end of the analysis pipeline. Other outputs are also provided: a detailed statistics table (csv format), cohesive ends or direct terminal repeats (fasta format), and the phage genome sequence reorganized according to termini positions and completed with extremity or repeats if needed. Finally, as a comparison with our method, we also implemented the method from Li *et al*.^18^ (Supplementary Data).

#### Availability and implementation

The PhageTerm software is provided as a command line tool (https://sourceforge.net/projects/phageterm), as a Galaxy Toolshed, and on a Galaxy web server (https://galaxy.pasteur.fr).

#### Phage *de novo* assembly

To determine the termini of an unknown phage, a first step of *de novo* assembly was required to obtain a reference sequence. To validate that this assembly step did not alter the result of the software, we assembled the six reference phages (lambda, HK97, T7, P1, T4 and Mu) *de novo* using SPAdes^21^ with standard options. When using a reference assembled *de novo* one should consider two possible caveats: (i) assemblers frequently introduce mistakes at the edges of contigs due to the presence of low abundance reads that do not correspond to the actual phage sequence. This usually results in a drop of coverage at the contig ends. In such cases PhageTerm should still be able to call the termini correctly, but we recommend correcting the contig ends when possible. (ii) In the case of phages with terminal repeats, most assemblers will output a contig with a single copy of the repeat, which is the desired input for PhageTerm. Note, however that some assemblers might create a contig with a repeat at each end. In such a case PhageTerm will incorrectly call multiple termini. We tested this hypothesis using the T7 reference genome (NC_001604, Table S1) and the PhageTerm output was “Multiple” on both strands for the two analysis methods.

#### Nucleotide sequence accession numbers

The complete genome sequences of phiCD24-1, phiCD111, phiCD146, phiCD481-1, phiCD505, phiCD506, phiMMP01, and phiMMP03 were deposited in European Nucleotide Archive under the accession no. LN681534, LN681535, LN681536, LN681538, LN681539, LN681540, LN681541, LN681542. The *de novo* assemblies performed on reference phage genomes are available at SourceForge (https://sourceforge.net/projects/phageterm). Phages used in this study are described in Table S1. The raw reads data of all phages were deposited in sequence read archive (SRA) under the accession number SRP093616 (Table S2).

## Results

Different mechanisms of DNA packaging can lead to a variety of DNA ends (Supplementary Table S4). When DNA ends occur at fixed or preferred positions along the phage genome, we expect to observe more reads starting at these positions than elsewhere in the genome (Fig. 1A). We re-sequenced six well-characterized *Escherichia coli* phages that use various packaging mechanisms (Lambda, HK97, P1, T7, T4, Mu) in order to validate our strategy. The PhageTerm analysis aligns the reads to a reference sequence provided by the user and computes the number of reads starting at each position (SPC), the sequence coverage (COV) and a variable *tau*: $\tau =SPC/COV\,\,$which displays several useful properties that allows classification of the phages (see materials and methods).

### Fixed DNA ends

#### COS

**Figure 2:**
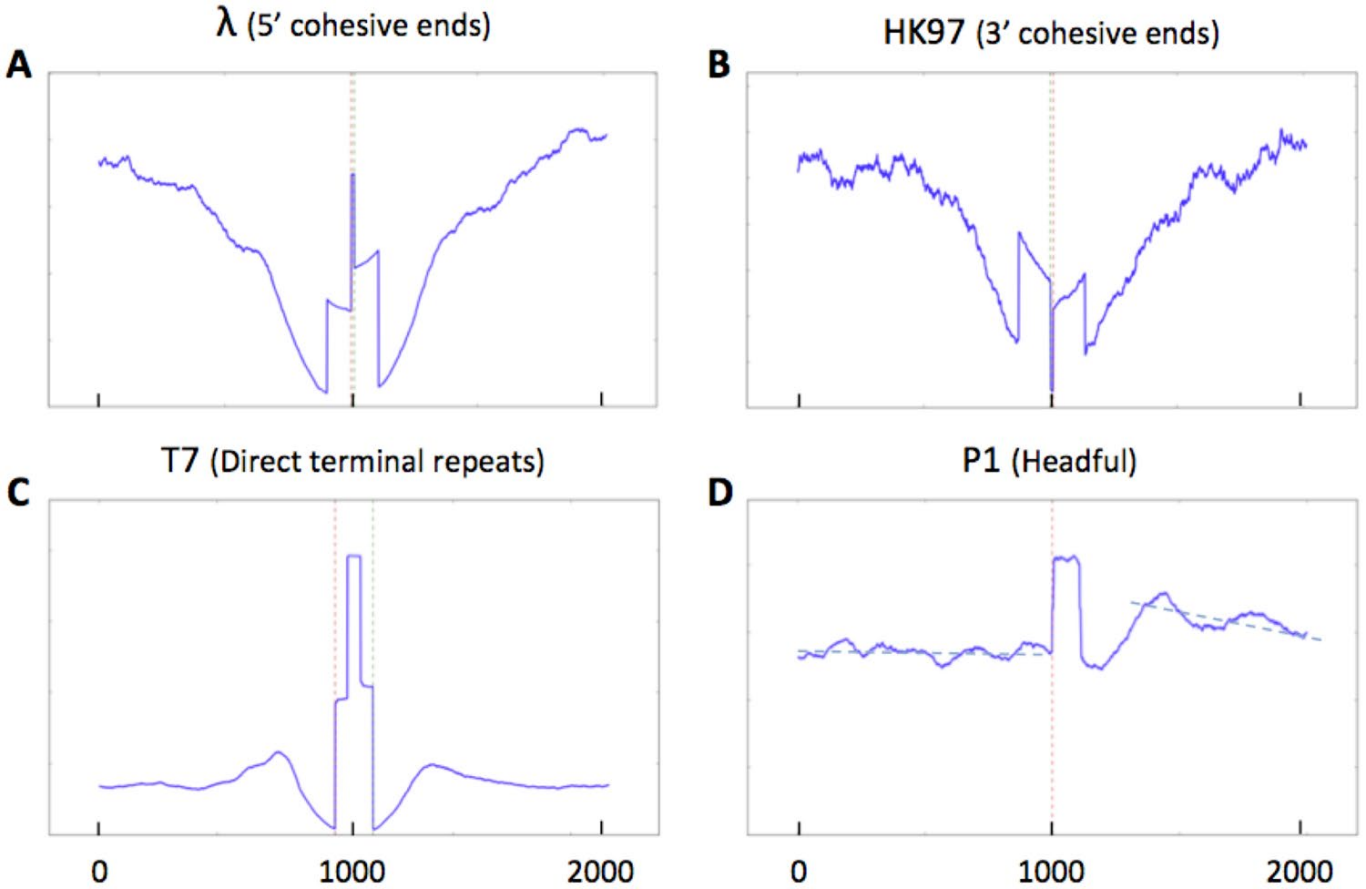
Sequence coverage at termini position. The sequence coverage around the termini identified by PhageTerm was plotted for the following phages: Lambda, HK97, T7 and P1. Exact termini positions are represented by dotted red line (Red: left; Green: right). Note the higher coverage obtained for phage P1 after the packaging site.

Cos phages are expected to display a single peak in each orientation with a value of 0.5 < *τ* < 1 (see materials and methods). These phages thus provide a very strong signal and can be easily identified. If the phage DNA sample was not contaminated with unpackaged DNA, no reads overlapping the *cos* site should be detected. As a result, *de novo* assemblers should naturally place the DNA ends at the contig limits. Note that different results are expected for *cos* phages with a 5′ overhang and those with a 3′ overhang (Supplementary Fig. S1). The enzyme typically used during the step of DNA end repair fills 5′ overhangs but degrades 3′ overhangs. As a result, phages with 5′ overhangs are expected to share the same terminal sequence over the length of the cohesive end, while phages with 3′ overhangs will have different terminal sequences. In practice, reads that cross the *cos* site are frequently recovered from contaminating DNA (prophage or circular forms), which can lead the assembler to place the *cos* site at a random position along the contig. In this case the peak positions will fall in close proximity to each other. On the one hand, *cos* phages with 5′ overhangs will have the forward peak positioned to the left of the reverse peak with the region in between showing twice the average sequence coverage (Fig. 2A). On the other hand, *cos* phages with 3′ overhangs are expected to have the forward peak to the right of the reverse peak, and the sequence in between is expected to have a very small coverage (Fig. 2B).

As an example of a 5′*cos* phage, we re-sequenced the well-characterized *Siphoviridae* coliphage Lambda^22^. The termini are known to consist of 5′ single-stranded cohesive overhangs of 12 bases. As a model for 3′*cos* phage, we re-sequenced HK97^5^. The PhageTerm software was able to determine the exact characteristics of Lambda and HK97: the nature of the termini (Fig. 1B), the *cos* mode of packaging and the length of their cohesive sequence (Table 1). PhageTerm was also able to accurately recognize phage Efm1 from Enterococcus as a 3′*cos* phage^23^. Among the *Clostridium* phages that we sequenced, phiCD481-1 and phiCD506 also matched the theoretical description of *cos* phages with 3′ overhangs (Table 2; Fig. 2B).

**Table 1:** Characteristics of reference phages.

Phage Name	Genome	Left	Right	Class	T/R	Fragment size
*Phages references*	(*G*)	(*τ*)	(*τ*)			*(F)*
Lambda	48 kb	0.59	0.41	COS (5′)	126	421
HK97	39 kb	0.51	0.60	COS (3′)	115	353
T7	39 kb	0.47	0.51	DTR (short)	594	468
P1	94 kb	0.20	—	Headful (*pac*)	101	494
T4	168 kb	—	—	−	—	463
Mu	36 kb	—	—	Mu-like	—	485

**Table 2:** Summary of PhageTerm results.

Phage Name	Ends	Left	Right	Class	Type
***Reference phages***					
Lambda	Non Redundant	10868	10879	COS (5′)	Lambda
HK97	Non Redundant	2047	2036	COS (3′)	HK97
T7	Redundant	7740	7899	DTR (short)	T7
P1	Redundant	54885	Distributed	Headful (pac)	P1
T4	Redundant	Random	Random	Headful	—
Mu	Non Redundant	32031	Random	Mu-like	Mu
***Phages of C. difficile***					
phiCD481-1	Non Redundant	67	55	COS (3′)	HK97
phiCD506	Non Redundant	44	32	COS (3′)	HK97
phiMMP01	Redundant	44435	Distributed	Headful (pac)	P1
phiMMP03	Redundant	52254	Distributed	Headful (pac)	P1
phiCD211	Redundant	122595	122972	DTR (short)	T7
phiCD146	Redundant	40603	Distributed	Headful (pac)	P1
phiCD505	Redundant	Multiple	Multiple	—	—
phiCD111	Redundant	Multiple	Multiple	—	—
phiCD24-1	Redundant	Random	Random	—	—
***Phages from SRA***					
T3	Redundant	38209	231	DTR (short)	T7
T7	Redundant	7740	7899	DTR (short)	—
Efm1	Non Redundant	1	42597	COS (3′)	HK97
PBES-2	Redundant	200	642	DTR (short)	—
HP1 (nextera)	Redundant	Multiple	Multiple	—	—
Slur09 (nextera)	Redundant	Multiple	Multiple	—	—

#### DTR

Phages with direct terminal repeats are expected to have one significant peak in each orientation, and the forward peak should be to the left of the reverse peak. The region between the peaks should show twice the average sequence coverage (Fig. 2C). Because of their terminal redundancy, DNA assemblers typically attribute the first base of DTR phages randomly. A typical example of this is phage T7, a *Podoviridae* that has exact short direct terminal repeats of 160 bases^20^. The PhageTerm software was able to determine the exact characteristics of T7: the nature of its termini (Fig. 1B), the termini positions, and the exact length of its direct terminal repeats (Table 1).

### Headful packaging

Phages using a headful packaging mechanism typically generate a concatemer containing several copies of their genome. During packaging a first cut is made at the packaging site (*pac* site), but the following cuts are made when the phage head is full, leading to variable positions. The expected value of *τ* ($SPC/COV)\,\,$can be computed as $1/(C+1)$, where C is the number of phage genome copies per concatemer (see material and methods). Because packaging is directional and no precise cut is made upon termination of packaging, a peak is expected only in a single orientation, which also informs us about the direction of packaging. In the region after this peak, where the second cut is made, a slight increase in coverage is expected, as part of this region will be present twice in many phage particles. Note that the signal obtained to determine the position of the *pac* site is *C* + 1 times weaker than that for *cos* phages (Fig. S3). This phenomenon is amplified by the fact that the cleavage position of the terminase at *pac* sites can be imprecise, leading to several possible termini. As an example of this packaging mechanism we re-sequenced phage P1, a promiscuous myophage. PhageTerm was able to determine the exact characteristics of P1: the nature of the termini (Figs 1B and 2D), the *pac* mode of packaging and an estimation of the average number of genome per concatemer (Tables 1 and 2).

Other phages have been determined to package their genome through a headful packaging mechanism but with no preferred packaging signal. The packaged phage genomes are circularly permuted with random termini. No signal can be recovered for this type of phage. As expected, PhageTerm wasn’t able to detect any termini when analysing T4, a *Myoviridae* phage of *E. coli* (Fig. 1B; Tables 1 and 2).

### Mu-like phages

Mu-like phages are temperate phages that amplify their genome through replicative transposition. During packaging, a first cut is made at a given distance from the phage end in the host genome. Packaging then proceeds through a headful mechanism with the second cut being made on the other side of the phage in the host DNA. The packaged DNA termini thus correspond to various fragments of the host DNA (Fig. S4). A specific analysis is performed to discover this type of packaging mechanism which relies on paired-end sequence information. We looked for hybrid fragments, i.e. pairs of reads where one read matched to the phage genome and the other read matched to the host genome. The expected theoretical proportion of hybrid fragments can be computed (see material and methods) and if at least half the expected number of such fragment are found, PhageTerm will determine the phage to belong to this class.

**Figure 3:**
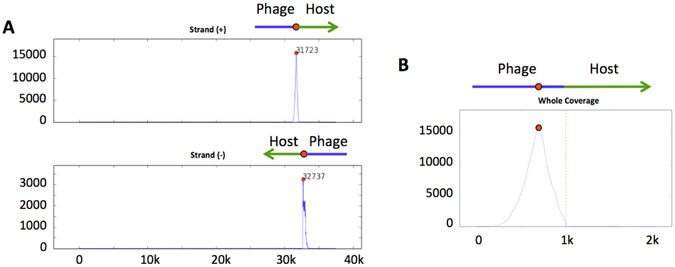
Detection of Mu-like phages. Hybrid fragments with one read on the phage genome and one read on the host genome are detected. (**A**) The sequence coverage of reads belonging to hybrid fragments of phage Mu is plotted along the phage genome. (**B**) Zoom in around the right terminus of phage Mu. The dotted red line represents the terminus position.

We re-sequenced phage Mu as the type-phage of this category. PhageTerm results were in line with those expected: no significant peak in the value of *τ* were found (Fig. 1B), and 2% of hybrid fragments were detected, in agreement with a theoretical expectation of 2.5% (Fig. 3). The host termini of Mu are asymmetric, with a short fragment on one side (~50 bp) and a long one on the other side (~2000bp). Unfortunately, the short read length provided by Illumina sequencing does not allow the determination of these fragment sizes easily and systematically.

### Peak size

Another interesting variable to consider when analyzing phage sequencing data is the number of reads starting at the termini (T) divided by the average number of reads starting at random positions (R). For phages with fixed termini, the size of the signal defined as *T*/*R* (R1 in Li *et al*.^18^) is expected to be equal to the average fragment size (F). Deviations from this expectation might indicate inefficient ligation of the sequencing adapters to the phage DNA termini, or the presence of other possible DNA termini. Sequencing results for phage T7 were in agreement with this prediction. However, *T*/*R* was markedly smaller than expected for phages Lambda and HK97 (Table 1). This was presumably due to an inefficient repair of the cohesive ends during our sequencing library preparation.

In the case of *pac* phages, an interesting observation is that knowing T/R and F, it is possible to estimate the number of phage copies per concatemer:

2 $$\,C=\frac{1}{\tau }-1=F\ast \frac{R}{T}-1$$

For phage P1 we can estimate the number of phage copies per concatemer to be ~4, in agreement with previous estimates^11^.

**Figure 4:**
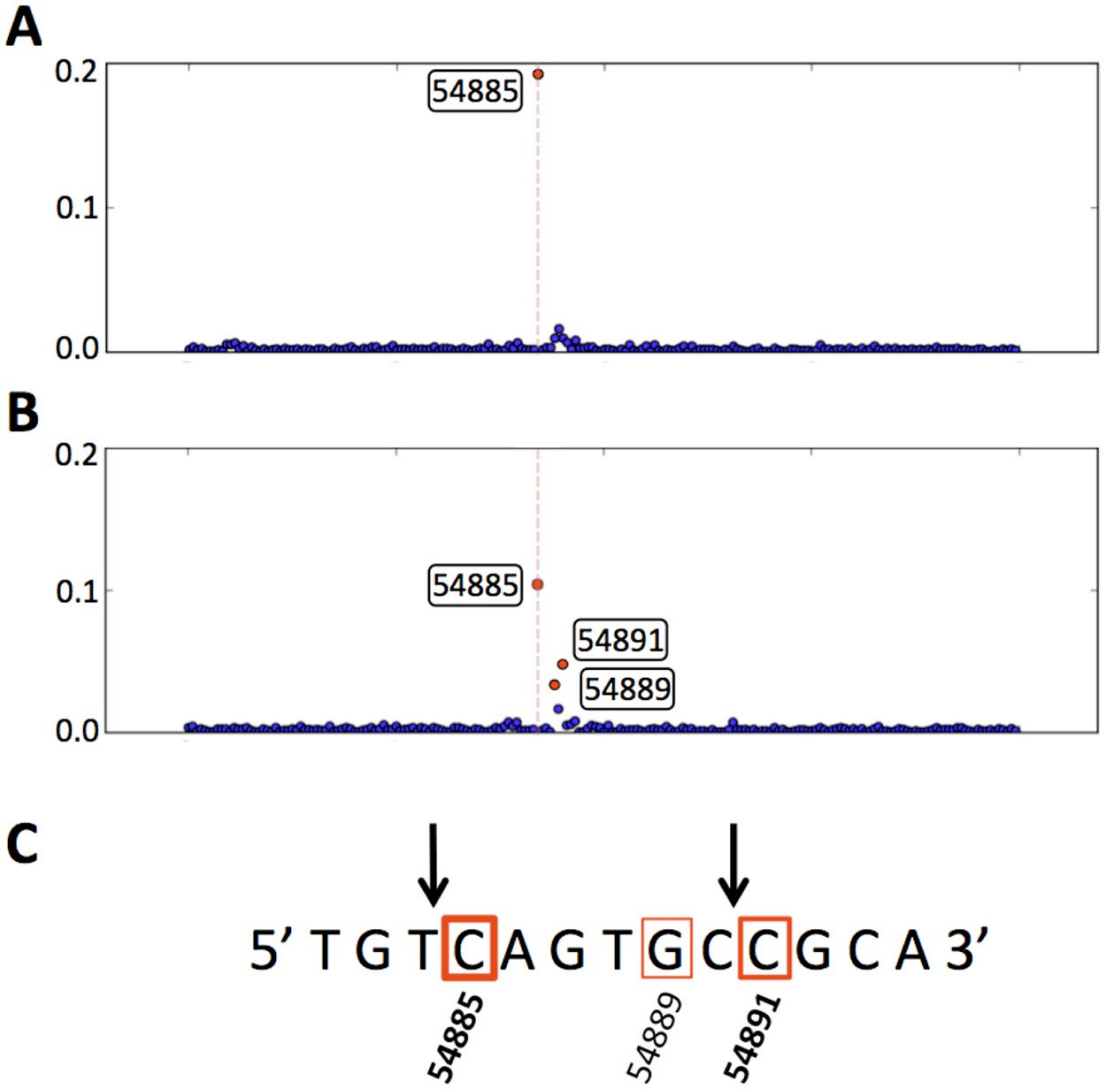
Bacteriophage P1 presence of secondary termini. Scatter plot of *τ* around the termini positions (**A**) with default surrounding option (20): one significant peak at location 54885 and (**B**) with surrounding option to 0: two close significant peaks appear. (**C**) Bacteriophage P1 DNA terminus region, arrows above represent the two main cleavage sites defined by Sternberg and Coulby in 1986, red boxes represent the significant termini found by PhageTerm.

Another aspect to be taken into account is the presence of secondary termini. These features appear when the site of cleavage by the terminase at the *pac* site is not precise^17^, which is the case for most headful phages analyzed to date^17^. By default, PhageTerm merges significant peaks close to each other into a single peak at position 54885 for phage P1 (Fig. 4A). However without merging (option «-d» was set to 0), two significant secondary termini could be found at positions 54885 and 54891 (Fig. 4B). Most significantly, the two main *pac* cleavage sites found for P1 were identical to the one determined by Sternberg and Coulby^24^ in 1986 (Fig. 4C).

### PhageTerm results

The PhageTerm software produces a detailed report containing several useful plots (coverage, SPC), a data table, information about termini types, and packaging mode. A sequence file beginning with the identified terminus is also generated and output. Some additional information is also extracted depending on phage characteristics: (i) an estimation of the phage concatemers size (pac phages); (ii) the sequence located between the two termini (*cos* and DTR).

#### Clostridium difficile Phages

*C. difficile* is currently the principal cause of antibiotic-induced infectious diarrhoea in humans^25^. Most *C. difficile* isolates analyzed to date carry one or several prophages; however, only a few of the prophages have been fully characterized. The packaging modes of four phages included in this study have been determined experimentally in our laboratory using the ligation-digestion method and were compared with the packaging modes predicted by PhageTerm. PhageTerm determined that phiCD481-1 and phiCD506 were *cos* phages, and found 13-nt 3′ overhangs for both phages (Table S1). These phages also showed the expected decrease in coverage between the termini of 3′*cos* phages (Fig. 2B). PhiCD146, phiMMP01 and phiMMP03 were found to be *pac* phages. PhiCD211 was found to have direct terminal repeats of 378 bp. No termini could be detected for phiCD505 and phiCD24-1, which could indicate a T4-like packaging mechanism. Finally, phiCD111 showed an atypical coverage plot, with decreasing coverage after a significant peak at the end of the genome, and another significant peak at another distant position. No packaging mechanism could be assigned to this phage.

#### SRA Phages

To test the versatility and robustness of the PhageTerm software on data generated by others, we have downloaded the raw sequencing data of six phages (T3, T7, HP1, Efm1, Slur09, and PBES-2) from the sequence read archive (SRA). Among these phages, four have known termini and packaging modes (T7, T3, HP1, and Efm1; detailed in supplementary table S1). PhageTerm was able to identify the correct termini and packaging modes for T3 and T7, along with exact short DTRs of 160 and 231 bases, respectively. For phage Efm1 and PBES-2, the PhageTerm software found a 3′ cohesive sequence of nine bases and direct terminal repeats of 443 bases, respectively.

#### User Phages

In the SourceForge deposit (https://sourceforge.net/projects/phageterm), we will also add interesting reports with user’s consent. One such example of an interesting report is that of Phi85, due to its long DTR.

All PhageTerm results are detailed in Table 2 and reports for all phages mentioned in this paper are available in the SourceForge deposit.

### Important note on sequencing library preparation methods

The methods described here rely on the random fragmentation of phage DNA and the availability of DNA termini to adapter ligation. As a consequence, methods that rely on transposases to ligate the adapters, such as Nextera, should not produce suitable data for PhageTerm and similar strategies^26^. We confirmed this by analyzing the data of phage HP1 and Slur09 that we retrieved from SRA. These phages were sequenced using Illumina Nextera kits, which led to the loss of the termini sequence. As expected no termini could be detected when using the data generated for these two phages.

### Transduction

Bacteriophages can also mediate horizontal gene transfer through transduction. During generalized transduction, a small number of viral particles can contain random fragments of host DNA, while during specialized transduction, only host DNA near the site of integration of the phage DNA can be transduced.

To test if phage capsids contained fragments of the host genome, the reads that did not match the phages were mapped to the host sequence, if provided. Note that the abundance of these reads only reflect transduction frequencies if the phage capsids were purified in a manner that excludes possible contaminations by host DNA. Phages P1 and Lambda were carefully purified to avoid such contaminations (Supplementary Data). P1 is known to cause generalized transduction while Lambda is not. As expected 3.8% of the reads obtained for P1 matched its host genome while only 0.01% of the reads obtained for Lambda did.

#### Specialized transduction

To test if phage capsids contained host DNA fused with phage DNA, we located the section of the hybrid fragments belonging to the host on the chromosome of the host. Peaks could be detected for all lysogenic phages at their attachment site in the host genome. The fragments detected could either come from specialized transduction or from host DNA contamination in the sample.

## Discussion

PhageTerm provides a simple interface for biologists to decipher phage termini and mode of packaging using NGS data. Our strategy relies on the analysis of the number of reads starting at each position along the genome divided by sequence coverage. This value, that we termed *τ*, has several informative properties that allow for the easy classification of phage types. This value is expected to be $0.5\le \tau \le 1$ for *cos* and DTR phages, and $\sim 1/(C+1)$ for *pac* phages where C is the number of phage copies per concatemer. Depending on the number of significant peaks that are detected, their size and orientation, it is possible to classify phages according to 6 types: 3′*cos*, 5′*cos*, short DTR, long DTR, headful with no *pac* detected, and headful with a *pac* site. An additional analysis of hybrid fragments carrying both phage and host DNA allows the identification of phages that amplify through replicative transposition (Mu-like phages). When no termini are detected and the phage cannot be classified as Mu-like, PhageTerm currently does not make any prediction of the packaging mechanism. In such cases, if the user is confident that the phage genome provided is complete, it can likely be classified as a T4-like phage. However, we cannot exclude other possible mechanisms such as a phi29-like packaging strategy^16^, which involves proteins covalently bound to the DNA termini. If proteins are not removed before adapter ligation during the sequencing library preparation, then we do not expect to obtain a signal with our strategy.

To compare our strategy with previous work, we also implemented the approach described by Li *et al*.^18^. In this work, the authors analysed the number of reads starting at each position along the genome and classified the phages according to the number and orientation of peaks, their size and the ratio of the first peak to the second peak. In most cases, both methods gave identical results, although PhageTerm performed a more fine-grained classification. A few cases highlight the benefits of using our method instead of the crude number of reads starting at the termini. *Clostridium* phage phiCD146 has a duplicated region that was collapsed during the *de novo* assembly. This can be seen as a sudden increase of coverage in this region. Li’s method assigned a wrong terminus in this high-coverage region, while PhageTerm was able to classify the packaging strategy of this phage. In another example, the *Clostridium* phage phiCD481-1 was correctly identified as a 3′*cos* phage by PhageTerm, whereas Li’s method mistakenly classified it as a *pac* phage, missing a peak in the reverse orientation where the coverage was unexpectedly low.

In previous work, other strategies have been developed to identify packaging mechanisms. In a recent publication by Rashid, J. *et al*.^27^ analyzing termini and packaging of *C. difficile* phages, researchers inferred packaging mechanism by looking at homologies between the terminase of newly-sequenced phages and that of phages with known packaging mechanisms. This strategy only provides partial and uncertain information about packaging mode and no information on termini type and sequences. Recently, another study made available a procedure to analyze whole genomes, physical ends and packaging strategies of phages using a pre-existing software^28^. This procedure can be very time-consuming and contains numerous steps requiring bioinformatic skills, including the installation of the Phamerator program^29^ and the management of the related SQL databases. Finally, a concomitant study^30^ predicted genome terminus by calculating two criteria, read edge frequencies and neighboring coverage ratio. Finding these procedures heavy and unwieldy for most phage scientists was that motivated us to develop PhageTerm as an easy-to-use program that consolidates into a single straightforward analysis numerous highly-valuable pieces of information about phage termini and packaging mode.

PhageTerm provides a rapid and reliable analysis of NGS data to determine phage termini and packaging mode, as long as sequencing data is generated following two simple rules: (i) random fragmentation should always be used when preparing sequencing libraries, and (ii) paired-end sequencing should be used to obtain a more complete characterization of the termini. Based on our data, PhageTerm outperformed other available phage analysis software by the accuracy of its analyses, its simplicity of its use, and the detailed reports it generates.

Increasing the availability of information on phage termini will shed light on the diversity of packaging mechanisms in nature. PhageTerm will assign termini regardless of whether it corresponds to a known phage packaging mechanism. As such, the software might help in the identification of novel packaging strategies. We encourage scientist community to contact us in the case of such discovery and we will be happy to update the software to better take into account any novel class. Finally, similar approaches should be possible to determine the termini of Archaea and eukaryotic viruses.

## Electronic supplementary material

Supplementary Data
