# Supplementary material for: PhageTerm: a tool for fast and accurate determination of phage termini and packaging mechanism using next-generation sequencing data
Source: Sci Rep. 2017 Aug 15;7:8292. doi: 10.1038/s41598-017-07910-5 (PMC5557969; doi:10.1038/s41598-017-07910-5)
Supplement: Supplementary file 1 — Supplementary Data [file 41598_2017_7910_MOESM1_ESM.pdf]

PhageTerm: a tool for fast and accurate determination of phage termini and packaging mechanism using next-generation sequencing data (Supplementary Data)

Julian R. Garneau[1,2], Florence Debardieu[3], Louis-Charles Fortier[2], David Bikard[3*] and Marc Monot[1,2*]

[1] Institut Pasteur, Laboratoire Pathogenèse des bactéries anaérobies, Département de Microbiologie, Paris, France.
[2] Département de Microbiologie et d'infectiologie, Faculté de Médecine et des Sciences de la Santé, Université de Sherbrooke, Sherbrooke, QC, Canada.
[3] Institut Pasteur, Groupe Biologie de Synthèse, Département de Microbiologie, Paris, France.
[4] Université Paris Diderot, Sorbonne Paris Cité, Paris, France.

Supplementary Methods

Phage DNA extraction and sequencing

***Escherichia coli* phages.** Phage Lambda, HK97, T7, P1 and T4 were propagated on E. coli strain MG1655 in LB supplemented with CaCl2 (5 mM). A thermosensitive variant of bacteriophage Mu (MuCts62) was induced from strain RH8816, a derivative of strain MC4100[1]. Strain RH8816 was grown at 30°C to OD~0.5 and induced at 42°C for 2 hours.

Phage capsids were isolated using a PEG precipitation step followed by cesium chloride (CsCl) gradient purification. First, solid NaCl was added to the phage lysate to a final concentration of 1M and incubated on ice for 1h, followed by centrifugation at 11,000g for 10 min at 4°C to remove the cellular debris. PEG8000 was added to a final concentration of 10% w/v for 1h on ice followed by centrifugation at 11,000g for 10 min at 4°C. The pellet was resuspended in PBS (1X) and incubated for 1h at room temperature. An equal volume of chloroform was then added followed by centrifugation at 3000g for 15 min at 4°C. The aqueous phase which contains the phage particles was recovered and further purified by ultracentrifugation with a gradient of cesium chloride (CsCl). A volume of 3 ml of each of two cesium chloride solutions (density of 1.3 g/ml- 0,41 g/ml of water and density of 1.5 g/ml- 0,68 g/ml of water) and a volume of 2ml of a CsCl solution (density of 1.6 g/ml- 0,82 g/ml of water) was added sequentially from more to less dense with a pipette into the bottom of a polyallomer tube. Phage suspensions up to 3 ml were then layered on the top of the gradient, and tubes were centrifuged at 100,000 g for two hours in a Beckman SW41 swinging bucket rotor. During this time, a roughly linear gradient of CsCl was formed in the tubes. Phages concentrated as a visible band were collected after puncturing the tube with a needle under the corresponding band. A second step of equilibrium ultracentrifugation in cesium chloride solution (density of 1.5 g/ml) was performed at 150,000 g with Beckman SW60 rotor. Phages were then dialyzed twice against water for 20 min and overnight against Tris 10 mM and NaCl 150 mM. Concentrated samples of phage lysates were treated with DNase and RNase for 30 min at 37°C, DNase was inactivated by EDTA (pH 8.0, 5 mM) for

10 min at 65°C and then proteinase K (0,5 mg/ml) and SDS (0,5 %) was added for 30 min at 55°C. Phage DNA was then purified using a typical protocol with phenol/chloroform/isoamylic alcohol followed by precipitation with acetate sodium (0,3M) and ethanol 100%. Pellets were washed with ethanol 70% and resuspended in TE (1X). Phage DNA was sequenced using the TruSeq library preparation kit on a HiSeq device.

***Clostridium difficile* phages.** The NGS sequences of 8 Clostridium phages are reported: three temperate *Siphoviridae* phages (phiCD24-1, phiCD111, and phiCD146), and five temperate *Myoviridae* phages (phiCD481-1, phiCD505, phiCD506, phiMMP01 and phiMMP03). Phage DNA was isolated from 10 mL of purified phage lysate using the QIAGEN Lambda Mini Kit as described previously[2]. Since no suitable propagating host could be identified, we extracted phage DNA directly from a crude induction lysate. The quality of the DNA preparations was verified on agarose gel and the absence of contaminating bacterial DNA was verified by PCR using primers targeting the triose phosphate isomerase gene[3]. Single-end multiplex TruSeq libraries were created according to Illumina's specifications. Sequencing was performed on the Illumina HiSeq 2000 platform of the Pasteur Institute (Paris, France). The 110 bp sequencing reads (12 to 19 Mb, >1000X) were assembled into a single contig for each phage using Velvet[4]. Finally we used the MicroScope workflow[5] to generate an automatic functional annotation for putative coding sequence (CDS), which was then manually curated.

Implementation of the method from Li *et al.*

This approach is based on the computation and interpretation of three specific ratios R1, R2 and R3. The first ratio, is computed as follow: the highest starting frequency found on either the forward or reverse strands is divided by the average starting frequency, R1 = (highest frequency/average frequency). Li's *et al.* have proposed three possible interpretation of the R1 ratio. First, if R1 < 30, the phage genome does not have any termini and is either circular or completely permuted and terminally redundant. The second interpretation for R1 is when 30 ≤ R1 ≤ 100, suggesting the presence of preferred termini with terminal redundancy and apparition of partially circular permutations. At last, if R1 > 100 that is an indication that at least one fixed termini is present with terminase recognizing a specific site. The two other ratios are R2 and R3 and the calculation is done in a similar manner. R2 is calculated using the two highest frequencies (T1-F and T2-F) found on the forward strand and R3 is calculated using the two highest frequencies (T1-R and T2-R) found on the reverse strand. To calculate these two ratios, we divide the highest frequency T1 by the second highest frequency T2. So R2 = (T1-F / T2-F) and R3 = (T1-R / T2-R). These two ratios are used to analyze termini characteristics on each strand taken individually. *Li et al.* suggested two possible interpretations for R2 and R3 ratios combined to R1. When R1 < 30 and R2 < 3, we either have no obvious termini on the forward strand, or we have multiple preferred termini on the forward strand (when 30 ≤ R1 ≤ 100). If R2 > 3, it is suggested that there is an obvious unique termini on the forward strand. The same reasoning is applicable for the result of R3, used to analyze the termini on the reverse strand. Combining the results for ratios found with this approach, it is possible to make the first prediction for the viral packaging mode of the analyzed phage. A unique obvious termini present at both ends (both R2 and R3 > 3) reveals the presence of a *cos* mode of packaging. The headful mode of packaging *pac* is concluded when we have a single obvious termini only on one strand.

Software implementation

PhageTerm is written in Python 2.7, the source code and documentation are freely available at https://sourceforge.net/projects/phageterm under a GPL license (GPL 3.0). PhageTerm is compatible on three operating systems (Linux, Mac OS X, and Windows) in command line (require Python 2.7.X with the following library: matplotlib 1.3.1, numpy 1.9.2, pandas 0.19.1, sklearn 0.18.1, scipy 0.18.1, statsmodels 0.6.1 and reportlab 3.3.0.). For direct graphical interface operation, a Galaxy wrapper version is available at https://galaxy.pasteur.fr.

Inputs and process

PhageTerm works with two mandatory inputs: (i) a file containing the phage sequence in Fasta format (Reference Sequence); and (ii) a file containing the sequencing reads in fastq format (Reads File). Optional inputs can also be chosen: the phage name to be used as an output prefix (default: Phagename), the length of the seed used for reads in the mapping process (default: 20), a surrounding value for peaks merging (default: 20), a file containing paired-end reads in fastq format (Paired File), the host genome sequence in fasta format (Host Sequence), phage upper limit coverage (default: 250) and the number of processor cores you want to use (default: 1).

Performance

The PhageTerm software was optimized to use multiple core and to minimize the memory consumption. During the whole process, the memory footprint remained low (< 1Gb). In our experiments, PhageTerm processed reads at a rate of more than 1 million reads per CPU-hour (3.5GHz Intel Xeon E5 with 12MB L3 cache).

How NGS reads quantity affect results ?

To test the reliability of the two methods, results were analysed with coverage values from 50 to 500 (Table S3). PhageTerm was able to accurately determine the termini of most phages down to a coverage of 50. Only a few *C. difficile* phages required a coverage of 250 or more to determine the right termini. PhageTerm can thus still perform very well with low sequence coverage.

Table S1. Phages used in this study

Name	Host	Genome	Accession	Terminus Type	Packaging
Lambda	*E. coli*	48 kb	NC_001416	5' Cohesive ends (12 bp)	COS
HK97	*E. coli*	39 kb	NC_002167	3' Cohesive ends (12 bp)	COS
T7	*E. coli*	39 kb	NC_001604	Short Exact direct terminal repeat (160)	COS
T3	*E. coli*	38 kb	KC960671	Short Exact direct terminal repeat (230)	COS
HP1	*H. influenzae*	32 kb	NC_000929	Cohesive ends (7 bp)	COS
P1	*E. coli*	94 kb	NC_005856	Headfull	PAC
T4	*E. coli*	168 kb	NC_000866	Headfull	PAC
Mu	*E. coli*	36 kb	NC_000929	Host DNA at termini	PAC
phiCD481-1	*C. difficile*	32 kb	NC_028951	3' Cohesive ends (13)*	COS
phiCD506	*C. difficile*	32 kb	NC_028838	3' Cohesive ends (13)*	COS
phiMMP01	*C. difficile*	44 kb	NC_028883	Headfull*	PAC
phiMMP03	*C. difficile*	52 kb	NC_028959	Headfull*	PAC
phiCD146	*C. difficile*	41 kb	NC_028958	Headfull¶	PAC
phiCD111	*C. difficile*	41 kb	NC_028905	-	PAC
phiCD211	*C. difficile*	131 kb	NC_029048	Short Exact direct terminal repeat (378)¶	-
phiCD505	*C. difficile*	49 kb	NC_028764	-	-
phiCD24-1	*C. difficile*	44 kb	LN681534	-	-
Efm1	*Enterococcus*	42 kb	NC_024356	3' Cohesive ends (9 bp)	COS
PBES-2	*Chronobacter*	149 kb	NC_028672	Short Exact direct terminal repeat (230)¶	COS
Slur09	*E. coli*	111 kb	NC_028840	-	-

**ligation-digestion (data not shown); ¶PhageTerm.*

Table S2. Phage Sequence information

Name	Machine	Library Kit	Reads	Assembly	SRA
Lambda	HiSeq	TruSeq	16 540 237	Spades	SRP093616*
HK97	HiSeq	TruSeq	87 747	Spades	-
T7	HiSeq	TruSeq	13 698 616	Spades	SRP093616*
T7	MiSeq	TruSeq	349 939	-	SRR831092
T3	HiSeq	TruSeq	100 000	-	SRR630513
HP1	MiSeq	Nextera	1 057 566	-	ERR966594
P1	HiSeq	TruSeq	13 172 521	Spades	SRP093616*
T4	HiSeq	TruSeq	14 133 503	Spades	SRP093616*
Mu	HiSeq	TruSeq	11 489 001	Spades	SRP093616*
phiCD481-1	HiSeq	TruSeq	18 176 892	Velvet	SRP093616
phiCD506	HiSeq	TruSeq	16 711 570	Velvet	SRP093616
phiMMP01	HiSeq	TruSeq	18 047 854	Velvet	SRP093616
phiMMP03	HiSeq	TruSeq	24 512 476	Velvet	SRP093616
phiCD211	HiSeq	TruSeq	12 607 885	Velvet	SRP093616
phiCD146	HiSeq	TruSeq	12 237 275	Velvet	SRP093616
phiCD505	HiSeq	TruSeq	13 977 881	Velvet	SRP093616
phiCD111	HiSeq	TruSeq	12 266 850	Velvet	SRP093616
phiCD24-1	HiSeq	TruSeq	19 326 652	Velvet	SRP093616
Efm1	HiSeq	TruSeq	1 154 818	-	-
PBES-2	MiSeq	TruSeq	77 472	-	SRR2121888
Slur09	MiSeq	Nextera	211 465	-	ERR1039470

**sequenced for this study*

Table S3. NGS reads quantity methods reliability.

Phage Name	Li's Method				PhageTerm Method				Limit
	500	250	100	50	500	250	100	50	
Phages references									
Lambda	✓	✓	✓	✓	✓	✓	✓	✓	50
HK97	-	-	✓	✓	-	-	✓	✓	50
T7	✓	✓	✓	✓	✓	✓	✓	✓	50
P1	✓	✓	✓	✓	✓	✓	✓	✓	50
T4	✓	✓	✓	✓	✓	✓	✓	✓	50
Mu	✓	✓	✓	✓	✓	✓	✓	✓	50
Phages of C. difficile									
phiCD481-1	✗	✓	✗	✗	✓	✓	✓	✓*	50
phiCD506	✓	✓	✓	✓	✓	✓	✓	✓	50
phiMMP01	✓	✓	✗	✗	✓	✓*	✗	✗	250
phiMMP03	✓	✓	✓	✗	✓*	✓	✓*	✓*	50
phiCD211	✓	✓	✓	✓	✓	✓	✓	✓*	50
phiCD146	✗	✗	✗	✗	✓	✓	✓	✗	100
phiCD505	✓	✓	✓	✓	✓*	✓*	✓	✓	50
phiCD111	✗	✗	✗	✗	✓*	✓*	✓*	✓*	50
phiCD24-1	✓	✓	✓	✓	✓	✓	✓	✓	50
Phages from SRA									
T3	-	-	-	✓	-	-	-	✓	50
T7	✓	✓	✓	✓	✓	✓	✓	✓	50
Efm1	✓	✓	✓	✓	✓	✓	✓	✓	50
PBES-2	-	-	✓	✓	-	-	✓	✓	50
HP1 (nextera)	✓	✓	✓	✓	✓	✓	✓*	✓	50
Slur09 (nextera)	-	✓	✓	✓	-	✓	✓	✓	50

* *after biological interpretation*

Table S4. Theoritical possibilities of phage genome in capsids.

Ends	Termini		Permuted	Extremity	Whole Coverage	Starting Position Coverage		Type
	Left (+)	Right (-)				Peak (strand +)	Peak (strand -)	
Non Redundant	Fixed	Fixed	No	5' overhang	Double between peaks	1 strong	1 strong	Lambda
				3' overhang	Very low between peaks			HK97
	Multiple	Multiple	Yes	-	-	Multiple strong	Multiple strong	-
	Random	Random	No	-	Hybrid insert*	no	no	Mu
Redundant	Fixed	Fixed	No	-	Double between peaks	1 strong	1 strong	T7
		Multiple			Double to the fist peak (-)		Multiple strong	-
		Preferred			Higher after peak (+)		1 weak	-
		Multiple-Preferred			Higher after peak (+)		Multiple weak	-
		Distributed			Increase at distributed range		no	-
		Random			-		no	-
	Preferred	Preferred	Yes	-	Increase between peaks	1 weak	1 weak	-
		Distributed			Increase at distributed range		no	P1
		Multiple-Preferred			-		Multiple weak	-
		Random			-		no	-
	Multiple	Multiple	Yes	-	-	Multiple strong	Multiple strong	-
		Preferred			Double from the last peak (+)		1 weak	-
		Multiple-Preferred			-		Multiple weak	-
		Random			-		no	-
	Multiple-Preferred	Multiple-Preferred	Yes	-	-	Multiple weak	Multiple weak	-
		Random			-		no	-
	Random	Random	Yes	-	-	no	no	-

Figure S1. COS phages with cohesive ends (e.g. λ - 5’, HK97 - 3’)

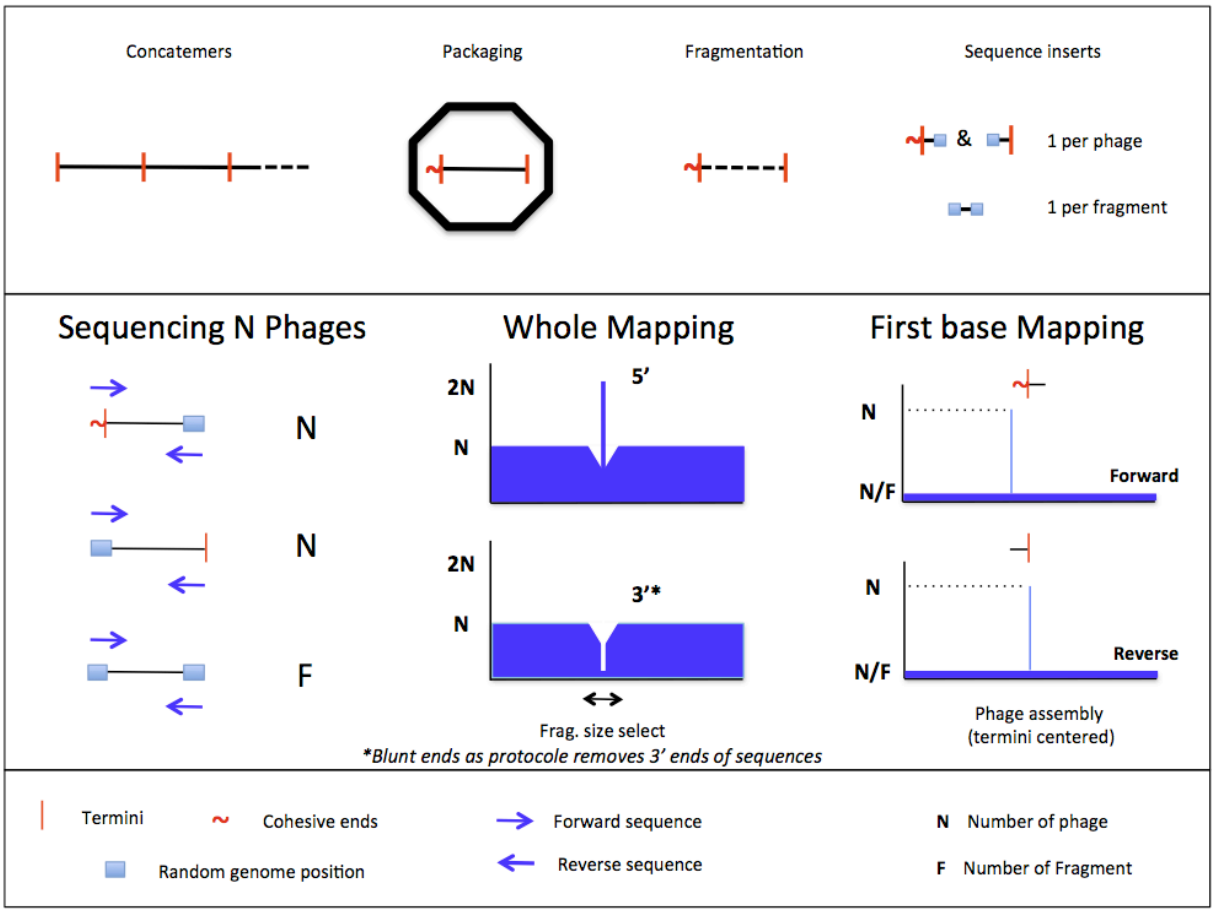

Figure S2. DTR Phage with direct terminal repeats (e.g. T7)

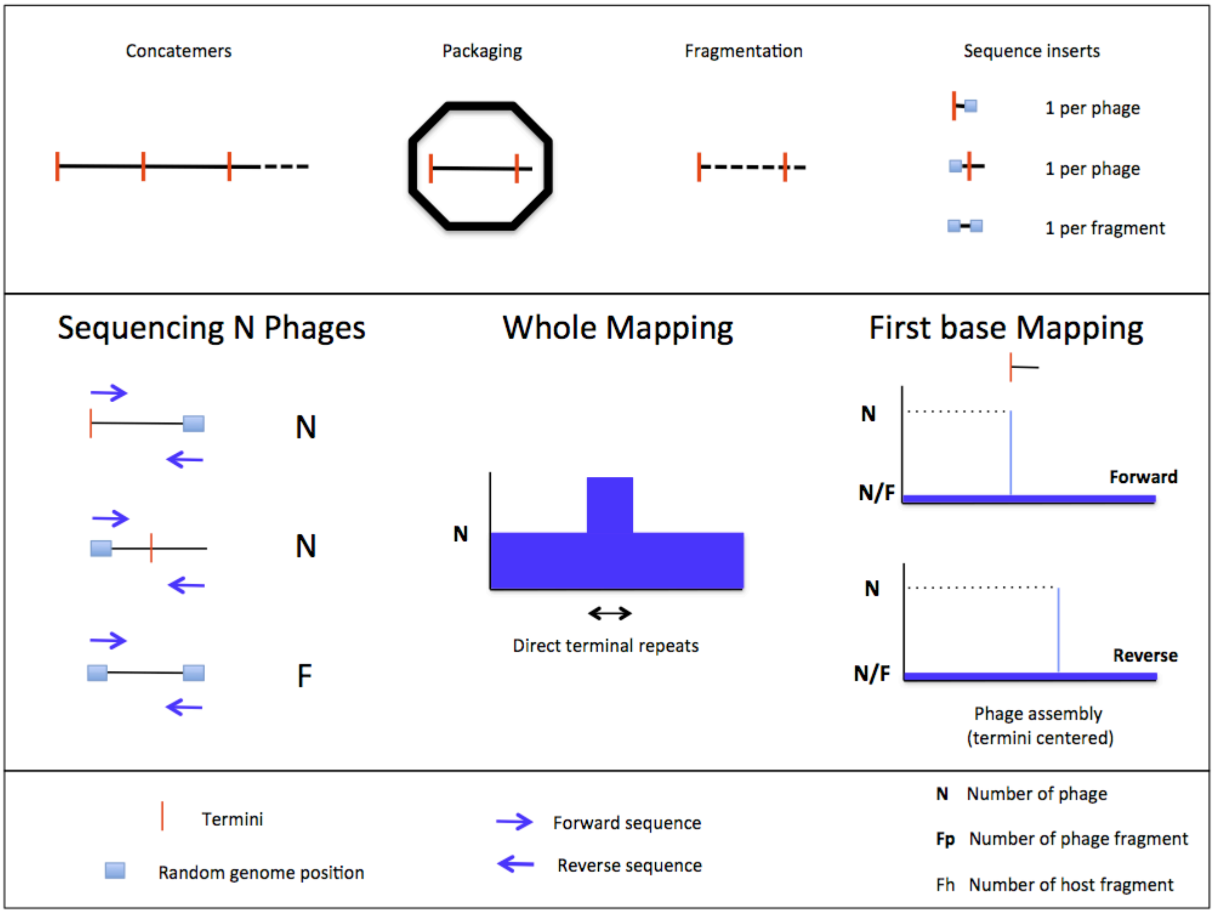

Figure S3. Headful phages with a prefered terminus (e.g. P1)

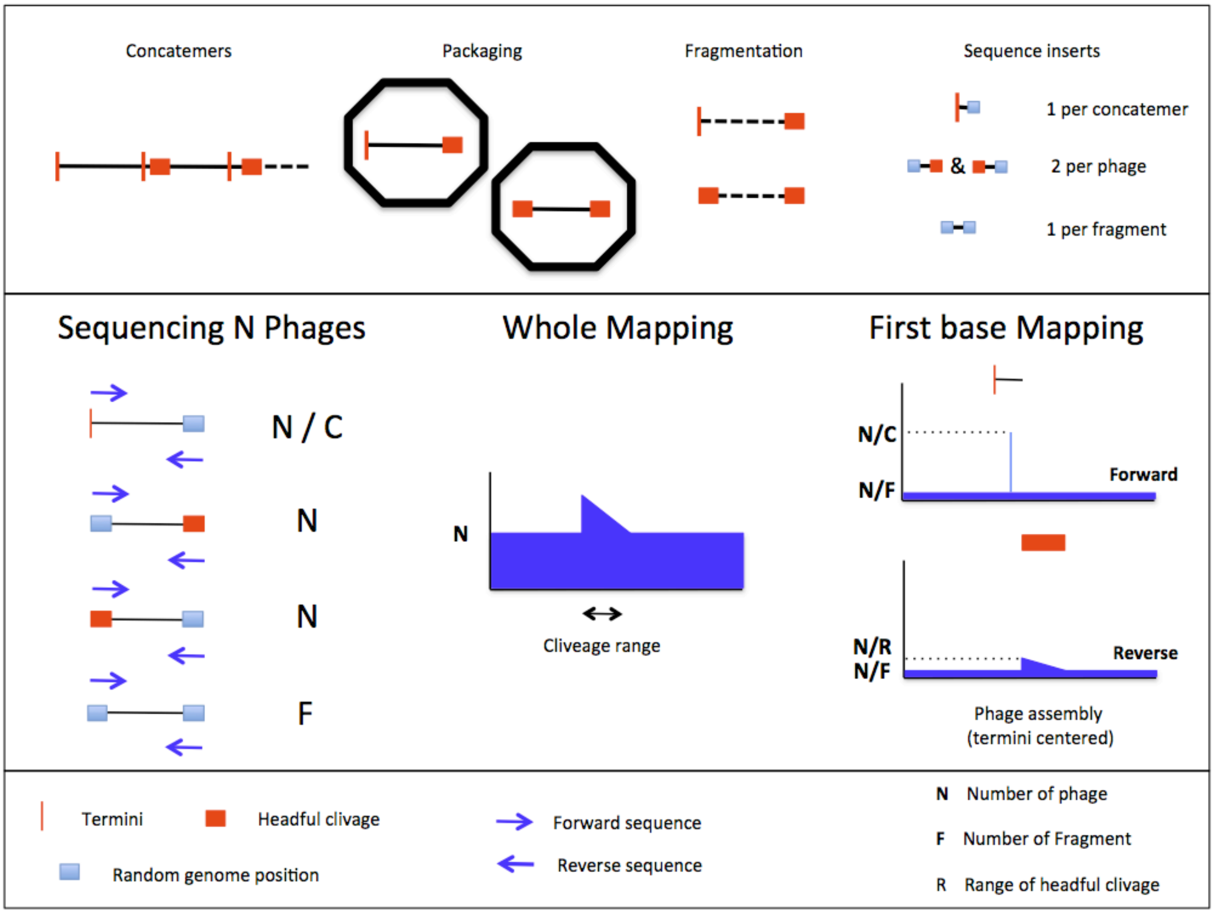

Figure S4. Mu-like phages with host termini (e.g. Mu)

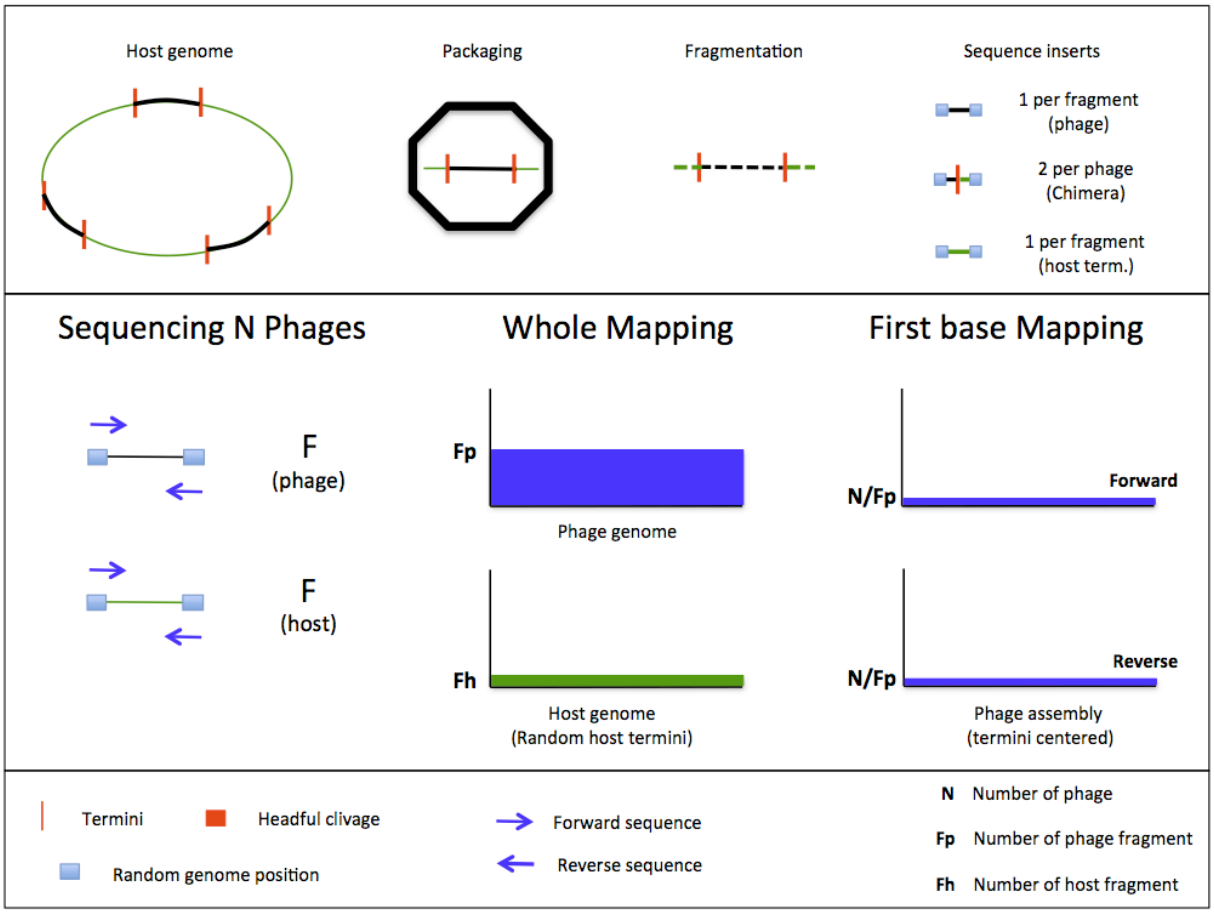

References

1 Vogel, J. L., Li, Z. J., Howe, M. M., Toussaint, A. & Higgins, N. P. Temperature-sensitive mutations in the bacteriophage Mu c repressor locate a 63-amino-acid DNA-binding domain. *J Bacteriol* **173**, 6568-6577 (1991).

2 Sekulovic, O., Meessen-Pinard, M. & Fortier, L. C. Prophage-stimulated toxin production in Clostridium difficile NAP1/027 lysogens. *J Bacteriol* **193**, 2726-2734, doi:10.1128/JB.00787-10 (2011).

3 Dhalluin, A. *et al.* Genotypic differentiation of twelve Clostridium species by polymorphism analysis of the triosephosphate isomerase (tpi) gene. *Syst Appl Microbiol* **26**, 90-96, doi:10.1078/072320203322337362 (2003).

4 Zerbino, D. R. & Birney, E. Velvet: algorithms for de novo short read assembly using de Bruijn graphs. *Genome Res* **18**, 821-829, doi:10.1101/gr.074492.107 (2008).

5 Vallenet, D. *et al.* MicroScope: a platform for microbial genome annotation and comparative genomics. *Database (Oxford)* **2009**, bap021, doi:10.1093/database/bap021 (2009).
